# Remarks on the Character and Writing of Hahnemann

**Published:** 1847-11

**Authors:** 


*Remarks on the Character and Writings of Hahnemann.* By the Editor of the New-York Journal of Medicine.

The September No. of the New-York Journal of Medicne contained a spirited article by its accomplished editor on the character and writings of Hahnemann, which has been issued by the publishers in a pamphlet form, in consequence of numerous applications for additional copies. The writer, in the first place, considers the Homœopathic system philosophically, and gives an analytical *expose* of its illogical errors, and its absurdities, founded on an examination of Hahnemann's publications. In the next place, he examines his character, as exemplified in his writings and personal history. Judging from the Homœopathic system alone, it requires no small exercise of charity, in a medical man, to believe that its founder was sincere and honest in its construction. In our estimation, as a system of philosophy or medicine, it is difficult to conceive of its having been engendered by folly only—there is a spice of knavery in it. That the same remark will apply to all who *profess* to practice medicine on this system we do not say. We are willing to admit that a person who has not received a medical education, and who is unfortunate in having but little intelligence, or a very ill-balanced mind, may, for a time, mistake the force of the *vis medi-*

*catrix* for the operation of infinitesimal doses. Perhaps we concede too much, but we would rather err on the side of charity. As regards converts from the ranks of the profession, if we were to address the Public, we should only say, who are they, what was their standing with their brethren, and were there no palpable inducements of a selfish or mercenary character to lead them to avail themselves of a popular delusion? A man of common shrewdness, if he will look around him, and put these questions to himself, can hardly fail to come to a correct conclusion. To return to Hahnemann, the circumstances mentioned in the following extract from Dr. Lee's article may be fairly considered as typical of the moral character of the man, and foreshadowing the development of his humbug on a larger scale:—

"In this early professional career (1800), it is admitted that he advertised a new salt, the discovery of which he claimed, and which he offered for sale under the name of *alkali pneum*, and the price of one *louis d'or* ($4) per ounce. The Society for the Promotion of Natural Sciences, at Berlin, procured an ounce of it from his agent at Leipzig, which was analyzed by Klaproth, and pronounced to be nothing more than *common borax!* Soon after this, Hahnemann adversised an "*infallible preventive of scarlet fever*," as he termed it; the price of which was, also, a *louis d'or;* this was a simple solution of the extract of *belladonna!* It has been proved by Professor Joerg, of Leipzig, that many of the quotations from old medical authors, made by Hahnemann, are false and fictitious! These statements, long since made, have never been refuted; it is admitted that his quackish practices chiefly drove him from Germany, to take up his abode in Paris.

The conclusions respecting the character of the founder of Homœopathy at which Dr. Lee arrives, are not dissimilar to those which we have expressed. He says:

"A careful examination of Hahnemann's works, then, compels us to believe that he was artful, disingenuous, and insincere. We doubt very much whether he himself believed in his own doctrines for years after they were first broached, and such was the opinion of some of his own intimate friends. His want of success in obtaining business in the treatment of disease, as well as the merited discipline to which he had been subjected, for violating the laws of his country, relating to the practice of medicine, had embittered his feelings against the regular profession, and against its modes of cure and led him to invent some antagonist system or school, which would present an opposing front to those whom he regarded as his enemies. Some of his friends, however, who judged him more leniently, believed that his object in inventing his theory of *similia similibus* was, that it lessened the evils which he saw to arise from excessive medication ; and if adopted, would leave the cure in the hands of Nature, in whose restorative energies he is said, by them to have had the most unlimited confidence. On either supposition, it follows that he was influenced by other motives than a belief in the truth of his doctrines.

Medical men are often asked why they do not give Homœopathy a trial, and the fact that they have not thrown aside legitimate practice, and tested the results of this system, is cited as evidence of their illiberality, and incompetency to decide on its merits. But what solid claims upon public confidence would the profession have, if its members were willing to make those who place health and life at their disposal, subjects for an experimental investigation of all the absurd systems which spring from the brains of dreaming enthusiasts, and cunning charlatans. Let those who are so ready to advocate every new humbug, make trial of the consequences in their own persons. Let them try lobelia, steam, and cayenne; Hydropathy; Homœopathy, and the various delusions, or impositions which are yet to be developed and supplant these; but let it not be asked of us to requite the confidence of those who place themselves in our hands to avail themselves of our best judgment and the resources of medical science, by exposing them to become victims to any form of delusion to which the proverbial credulity of a portion of mankind has given for a season a fashionable currency.

They who thus attack us seem to think that medical knowledge is exclusively, in the philosophical sense of the term, empirical—that is derived solely from blind experiments of the *laedentia et juvantia ;* and that we have no settled principles of pathology and therapeutics by means of which we can rationally estimate new propositions in these branches of science. We quote the remarks of Dr. Lee on this point, which present the subject in a strong, but true light:—

"The profession is constantly assailed by the followers of Hahneman for rejecting his doctrines without subjecting them to trial. Although not true to the extent alleged, we contend that the educated physician is justified in rejecting homœopathy without testing it at the bed-side ; and this opinion is founded on the fact, that its doctrines are not only contradictory and at variance with themselves but self-evident absurdities. If the medical man, who seriously sets about their verifications, does not endanger his reputation for soundness of mind, he, at any rate, compromits his character, as a thoroughly-educated physician, and a man of well-balanced intellectual faculties. Besides, it is unnecessary, an act of supererogation, to undertake the establishment of statements, which can be disproved by their opposition to facts already known, as well as their contradiction of one another. What would be thought of a man who should undertake to demonstrate that the three angles of a triangle are greater than two right angles; or that two and two make five; or that a part is greater than the whole? What must be thought of him who believes that a grain of medicine can be so expanded as to fill a space larger than the solar system, and yet that the bulk of a mustard seed retains sufficient power to affect sensibly the animal organism? Can a man believe an absurdity like this, and not need a dose of hellebore? The distance between the earth and the sun is 95,000,000 of miles; twenty homœopathic globules, laid side by side, extend to about an inch, so that 158,000,000,000 such globules would reach from the earth to the sun. But when the 30th (X) dilution is practiced, each grain is divided into 100,000,-000,000,000,000,000,000,000,000,000,000, 000,000,000,000,000,000,000-000,000 parts, so that a single grain of any substance, in the 30th dilution, would extend between the earth and the sun, 1,262; 626,-262; 626,262; 626,262; 626,262; 626,262; 626,262; 626,262; 626,-262 separate times! Can a system of medicine which puts forth such statements as this, be worthy of serious investigation? The medical man who is at all accustomed to examine, weigh, and compare facts, is justified, we say, in rejecting, at once, without examination, a system made up of such gross absurdities and fallacies. He is bound to do it in justice to his own understanding."

That Homœopathy has been of service may be conceded. The benefits, however, which have flowed from it, can, of course, only be appreciated by those who properly appreciate the system—i. e., by those who deem it, when honestly practiced, to be utterly nugatory in its effects. The benefits consist in the opportunities which it has afforded for the observation of disease uninfluenced by remedies, in cases in which we should be unwilling, nor, should we have been justified in abstaining from remedies, in order to determine what the unaided powers of life are capable of effecting. We shall do well to make the most of these opportunities, for, from present appearances, they will not long continue. The doctrine of *similia similibus*, and *infinitesimal quantities* has already passed the zenith of its popularity, and will soon be remembered only as one of the thousand fantastical absurdities which have had their day, imposing heavy penalties for the lessons of wisdom which they inculcate. We give, in conclusion the closing paragraph of our learned friend's interesting and able article:—

"Homœopathy, however, has not been productive of unmixed evil; for it has taught us the wonderfully restorative powers of Nature, when aided by the enforcement of rigid dietetic rules; and it has thus lessened the amount of active medicines given. Assuming, as a basis, hypothetical facts, which later experiments have disproved, proceeding on analogies which are dissimilar, involving contradictions irreconcilable, homœopathy has, at every stage of its progress, made war upon oommon sense, drawn largely upon human credulity, violated all the rules of philosophy, and has now settled dowd into that slough of contempt, from which its ablest advocates can never succeed in elevating it.

"*Requiescat in pace!*"

